# Continuance intention and digital health resources from the perspective of elaboration likelihood model and DART model: a structural equation modeling analysis

**DOI:** 10.3389/fpubh.2024.1416750

**Published:** 2024-06-14

**Authors:** Chengcheng Fei, Haixia Zhou, Wei Wu, Longyuan Jiang, Yuanqi Xu, Haiyan Yu

**Affiliations:** School of Medical Humanities and Management, Wenzhou Medical University, Wenzhou, China

**Keywords:** digital health resources, continuance intention, elaboration likelihood model, structural equation model, DART model, user value co-creation behavior

## Abstract

**Background:**

Internet hospitals, online health communities, and other digital health APPs have brought many changes to people’s lives. However, digital health resources are experiencing low continuance intention due to many factors, including information security, service quality, and personal characteristics of users.

**Methods:**

We used cross-sectional surveys and structural equation modeling analysis to explore factors influencing user willingness to continue using digital health resources.

**Results:**

Information quality (*β* = 0.31, *p* < 0.05), service quality (*β* = 0.19, *p* < 0.05), platform reputation (*β* = 0.34, *p* < 0.05), and emotional support (*β* = 0.23, *p* < 0.05) have significant positive effects on user value co-creation behavior. Additionally, user trust and perceived usefulness could mediate the association between user value co-creation behavior and continuance intention, with mediation effects of 0.143 and 0.125, respectively. User involvement can positively moderate the association between user value co-creation behavior and user trust (*β* = 0.151, *t* = 2.480, *p* < 0.001). Also, user involvement can positively moderate the association between value co-creation behavior and perceived usefulness (*β* = 0.103, *t* = 3.377, *p* < 0.001).

**Conclusion:**

The keys to solving the problem of low continuance intention are improving the quality and service level of digital health resources, and promoting users’ value co-creation behavior. Meanwhile, enterprises should build a good reputation, create a positive communication atmosphere in the community, and enhance user participation and sense of belonging.

## Introduction

With the gradual increase of Internet integration into the lives of residents, digital health resources such as Internet hospitals and online health communities have emerged (1). Internet hospitals, a common resource, use Internet tools to extend diagnosis and treatment services from within the hospital to outside the hospital. This overcomes time and space constraints and promotes the rational use of high-quality medical resources so that people can enjoy such services as online appointment booking, online diagnosis, and medicine delivery (2).

The online health community is another type of digital resource, involving health-related activities shared by a group of users with common health needs. These communities can be categorized based on types of users and communication modes into doctor-doctor, doctor-patient, and patient–patient online communities (3). Online health communities provide users with counseling and consultation, personal health management, and sharing of user information and experience, as well as health information in the form of video, audio, and graphics. These communities can meet the diverse health service needs of the population while saving user costs and optimizing the allocation of medical resources (4). However, problems have arisen in the development of digital health resources. Although people enjoy the convenience of the Internet, they tend to leave their data accessible in browsers and face the risk of information leakage or misuse. This greatly reduces trust and willingness to use digital health resources (5). Additionally, the massive amount of information on the Internet has caused information overload for users (6). Further, in online health communities, much of the information is published, disseminated, evaluated, and utilized by people with average levels of expertise, information literacy, and rationality (7). Therefore, the accuracy, scientific validity, and reliability of the information may be affected, and may even lead to misdiagnosis and inappropriate treatment advice, which in turn triggers anxiety in users (8). Other problems are that some users are not sure how to use digital systems for the first time, or they give up continuing to use them due to the interface being difficult to operate and the price of the service not being transparent (9). Current research focuses more on the willingness to use, while scholars have not yet explored how to mobilize the willingness to continue using, a concept known as continuance intention.

Current research on continuance intention mainly involves fields such as the Learning community (10–12), tourism business (13, 14), and information dissemination (15, 16). Not as much research has focused on the field of digital health, so our study aimed to fill that void. We aimed to explore and demonstrate the influence factors of continuance intention. Then, we combined different models from economics and information communication, using scenario-based modeling and structural equation modeling to discern reasons for willingness to continue the use of digital health resources. We explored the formation mechanism of continuance intention from multiple aspects, including information quality, platform quality, corporate reputation, and user experience. We aimed to supervise the development of the digital health industry, improve the user trust of the digital health and promote the using of digital health resources and promote the residents’ health status finally.

## Literature review and hypotheses

### Elaboration likelihood model

The elaboration likelihood model is the most influential model in recent years in the study of consumer information processing and persuasion. The theory distinguishes two methods based on the depth of information solving: the central and peripheral routes (17). When information receivers have high information processing motivation and ability, they tend to adopt the central route, in which they form rational judgments about target behaviors by examining the arguments, merits, and relevance of the issues. Conversely, when information receivers have insufficient motivation or ability to process information, they are more likely to adopt the peripheral route in which they use peripheral cues, such as the authority of the information source, to form a choice (18). The main variables of the center route include information quality or content quality (19), and the main variables of the peripheral route include source credibility, electronic word-of-mouth, and emotional support (20).

Information quality relates to the persuasive strength of the evidence embedded in the message. Drawing on the definition proposed by scholars (21), our analysis defined information quality as the extent to which a physician’s response to a patient’s message helps the patient to recognize the disease. Studies have shown that information quality directly influences recipients’ attitudes toward specific behaviors and messages (22). Therefore, information quality is an antecedent variable of users’ attitudes and behavioral intentions (23).

Informativeness and readability are two commonly identified dimensions of information quality. Readability is the extent to which information is easy to understand and can be measured using the average number of sentence words or characters, with larger values indicating more complex and difficult information to understand (24). Informativeness involves the amount of information provided in response to a question and is an significant antecedent variable that influences the adoption behavior of message recipients (25).

Service quality is another major variable of the central route and refers to the sum of features and characteristics that satisfy consumers’ stated and potential needs (26). For our analysis, we defined service quality as the reliability, timeliness, and satisfaction of all digital health resource-related services. Online consultation facilitates doctor-patient communication, and the attitude of the doctor during online consultation directly affects the user’s experience and willingness to interact; the more attentive the doctor’s service, the more likely it is that the user will be willing to interact with the Internet hospital (2). At the same time, the timeliness and matching of services received by users in the health community affects their behavioral choices.

Value co-creation behavior refers to the interaction and resource integration between users and enterprises to achieve common interests (27). In terms of digital health resources, UVCB is manifested in the willingness of users to bear the risks and responsibilities of using online health services. Users take the initiative to request that enterprises disclose information, actively participate in knowledge sharing and communication, and actively offer advice to enterprises and others (28).

*H1*: Information quality positively affects user value co-creation behavior.

*H2*: Service quality positively affects user value co-creation behavior.

Peripheral routes contain meta-information that is not included in the message from the source. When the receiver of the information lacks the ability and motivation to tackle the information, they are more inclined to use edge cues to aid in decision-making (19). Information such as corporate reputation and the emotional support of users are often identified as peripheral cues in the elaboration likelihood model (29, 30). For our study, corporate reputation specifically refers to positive public praise and reputation of a digital health resource. Some researchers showed that positive evaluations (reputation) of a product or service improve the customers’ evaluation of the service brand, whereas a negative reputation makes the consumer avoid the brand outright (31). When people use digital health resources, they look for information about the reputation of the company providing the service. If a firm’s reputation is more positive, the advice that users receive from it is more likely to influence their behavior (32). Also, emotional support, such as encouragement and caring that users receive when interacting with health service providers and with other community members, can influence users’ behavioral choices.

*H3*: Corporate reputation positively affects user value co-creation behavior.

*H4*: Emotional support positively affects user value co-creation behavior.

### DART model

Prahalad’s DART (dialog, access, risk, transparency) model states that users can co-create value with the enterprises with which they interact (33). This contrasts with the traditional model, where value creation is the sole responsibility of the provider or enterprise. Dialog refers to the communication and knowledge sharing between consumers and enterprises, and access refers to the fact that consumers do not need to own or purchase product experience and information, as this can be obtained through negotiation. Risk assessment refers to the fact that enterprises should give consumers more information about the products and services, and also need to encourage consumers to take on more responsibility. Transparency means that prices and costs must be publicly available, and it is not a solution for companies to obtain high returns through information inequality.

Trust is defined as a belief or attitude that simultaneously enhances the user’s perception of a service’s potential benefits and reduces the perception of the risks, thereby increasing the user’s willingness to purchase or adopt the service (34). Trust is an important factor influencing users’ adoption of a digital health resource (35), and includes users’ trust in Internet hospitals, doctor-patient trust, as well as trust in other members and information in an online health community. When using a digital health resource, if the interface is simple and easy to use, the data security is high, the risk is low, and the price information is transparent, users are more willing to interact with the software or web page and their trust in the platform will be enhanced (36). Positive feedback, such as thanks given by other users, can increase users’ sense of self-worth and thus deepen their trust in the usefulness of interactive communication (37). Also, when people trust online healthcare communities, their willingness to use them increases (38). Individuals tend to seek medical advice and support when they face health problems. The more trust they have in an online health community, the more likely they are to seek help in the community and the more likely they are to accept medical advice from the community (39).

Perceived usefulness means people believe that choosing a particular system or technology improves them, and is reinforced when either intrinsic needs are generated or direct external incentives are stimulated (40). The information users receive when interacting with other online health community members facilitates the speed of decision-making in choosing the right health service for them, thus enhancing their perceived usefulness of digital health resources (41). At the same time, the perceived usefulness of a product affects people’s willingness to continue using it, and when they perceive that a product is easier to use and more beneficial to them, their action intention is biased toward accepting it (42). Bhattacharjee confirmed the positive effect of the perceived usefulness of an information system on users’ intention to continue using it (43). Based on current knowledge about user trust and perceived usefulness, we hypothesized that they are mediating factors in user value co-creation behaviors, as follows.

*H5*: User trust mediates the association between UVCB and continuance intention.

*H6*: Perceived usefulness mediates the association between UVCB and continuance intention.

An information receiver’s reliance on either central routes or peripheral routes in decision-making is moderated by their mood to process information. Motivation is often measured as enrollment (30), a inner psychological status that reflects the degree of personal relevance and significance of a particular issue, advertisement, product, or task to the information receiver (22). For this analysis, we defined involvement as the perceived importance and relevance of a digital health resource. Existing research suggests that higher levels of enrollment indicate more demand for healthcare services and online health communication, and a greater likelihood of engaging in value co-creation behaviors for digital health resources, thereby strengthening the users’ trust (44). In Internet hospitals, when doctors make directed consultation suggestions, users with higher levels of involvement spend more cognitive and behavioral effort processing the doctors’ responses. This enhances users’ feelings of self-achievement and involvement, significantly strengthening their perceived usefulness of digital health resources (45).

*H7*: User enrollment positively moderates the association between the UCVB and user trust.

*H8*: User enrollment positively moderates the association between user UVCB and perceived usefulness.

**Figure 1:**
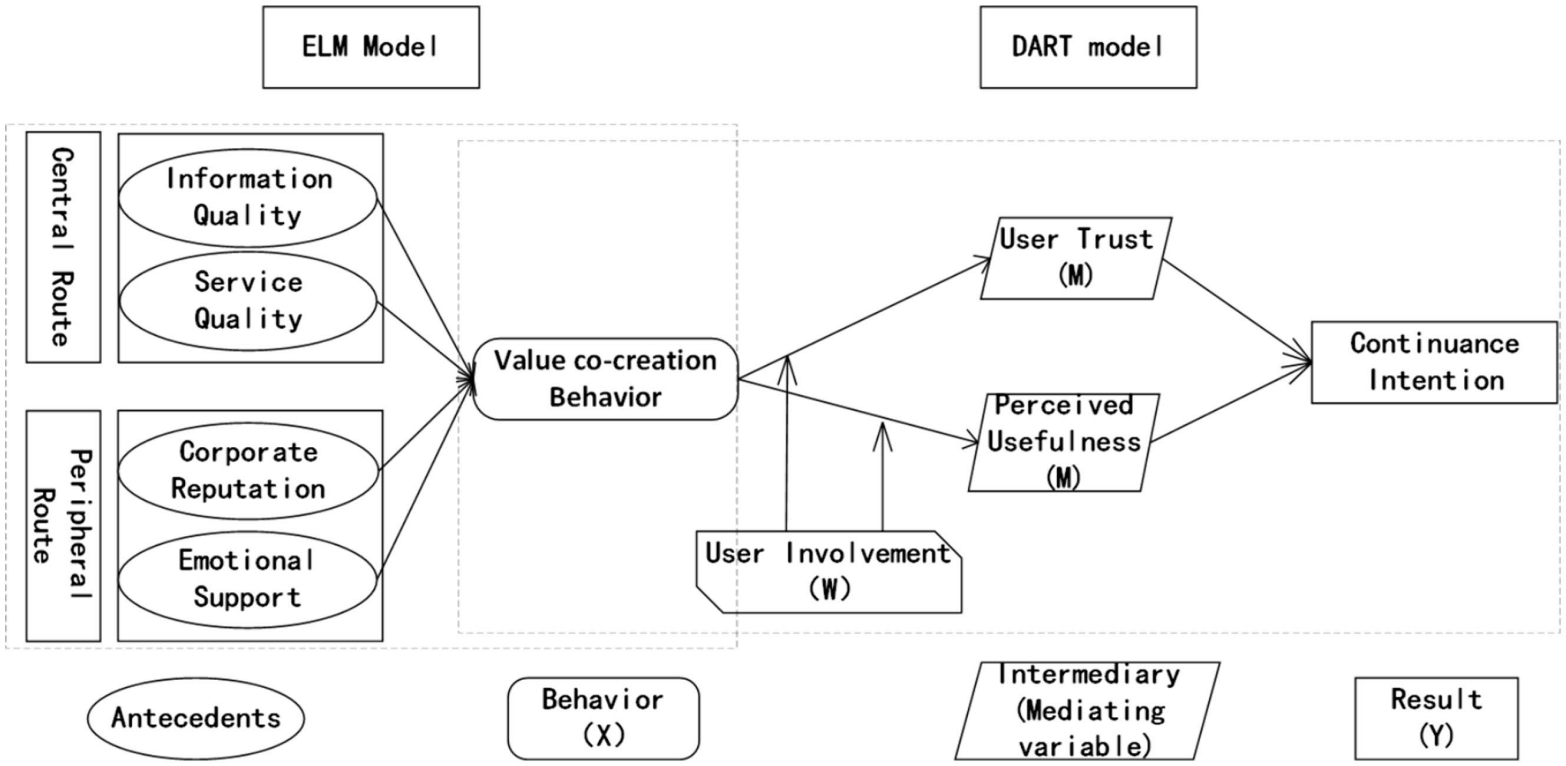
Research model.

Figure 1 is a diagram of our proposed hypotheses, using the theoretical logic of “antecedent-behavior-mediator-result.” The model represents service quality and information quality as dimensions of the central route, while platform reputation and emotional support are dimensions of peripheral route. These two routes determine how users engage in UVCB. UVCB is the independent variable and continuance intention is the dependent variable, with user trust and perceived usefulness as the mediating variables, and user involvement as the moderating variable.

## Methods

For this study, we invited residents of Jiangsu-Zhejiang-Shanghai District to answer a survey during the period October to November 2023. The questionnaire was revised and improved based on previous studies (46–50) and consisted of 33 items, including nine variables: information quality, service quality, platform reputation, emotional support, user value co-creation behavior, perceived usefulness, user trust, user involvement, and continuance intention. The responses were all tested using the Likert Level 5 scale. Before starting the formal survey, we conducted a presurvey and preliminary testing of the questionnaire items and conducted reliability and validity tests. Then, we carried out large-scale data collection. We randomly selected 10 sampling community from Zhejiang Province, Jiangsu Province, and Shanghai, and commissioned local mental health centers to cooperate in distributing questionnaires. People who had no experience using online psychological platforms were excluded from the survey. We trained all investigators and guided participants. The participants were told about the purpose of the research. The survey was anonymous, and all people signed informed consent forms before beginning the investigation. The study had got the approval by the Ethics Committee of Wenzhou Medical University, and all the information is for research purposes only.

We obtained 400 questionnaires and after excluding invalid questionnaires, we retained 373, with an effective rate of 93%. Then, we sorted and analysed the data. SPSS 26.0 was used to complete the descriptive statistics, moderation effects tests and Bootstrap tests. *p*-value<0.05 is the statistical significance. AMOS 26.0 was used to build the structural equation model and demonstrate the hypothesis. Structural equation model (SEM) is a confirmatory modeling technique used to investigate how well a hypothetical model fits the data (51). SEM can simultaneously estimates association between multiple variables. In this research, we have 9 variable and 8 hypothesis. If we use the traditional multiple regression method, we need to repeat the statistical process for many times, which will be quite cumbersome and complex. So we choose the SEM method to demonstrate the hypothesis.

## Results

### Descriptive statistics

Table 1 shows the basic information. Among the 373 participants, there were 174 males (46.6%) and 199 females (53.4%). From the age distribution of the respondents, it can be seen that the proportion of young people was relatively high, with the age group of 21–30 years old accounting for the highest proportion (43.2%). According to the distribution of educational qualifications, 61.9% of people received higher education at or above university level; 23.3% of participants frequently used (two or three times a week) digital health resources, while 7.5% of participants rarely used digital health resources. Among all digital health resources, 55.2% of users frequently use health consultation and knowledge learning functions; 42.8% frequently use online registration and appointment service; 45.6% frequently use online diagnosis and treatment services; 33.8% frequently search for disease-related information; 31.1% frequently use health and wellness services; 28.7% utilize digital resources for self-health management; and 22.8% frequently communicate and interact with other users online.

**Table 1:** Descriptive statistical analysis of subject demographics.

Information	Classification	Frequency	Percentage
Sex	Male	174	46.6
	Female	199	53.4
Age	Below 20	43	11.5
	20–30	161	43.2
	30–40	86	23.1
	40–50	54	14.5
	Over 50	29	7.8
Education	Middle school	62	16.7
	High school	80	21.4
	College/University	159	42.6
	Master degree or above	72	19.3
Frequency of using digital health resources	Almost every day	39	10.5
	2–3 times a week	87	23.3
	2–3 times a month	148	39.7
	2–3 times a year	71	19.0
	Rarely	28	7.5

### Common source variance test

This study selected the Harman’s univariate method to test for common source variance test. As we can see in Table 2, the maximum variance explanatory rate of the unrotated factor is 37.236%, which is lower than 40%. This indicates that the data has passed the common source variance test.

**Table 2:** Common source variance test.

Component	Initial eigenvalue			Extraction sums of squared loadings		
	Total	% of variance	cumulative %	Total	% of variance	cumulative %
1	10.426	37.236	37.236	10.426	37.236	37.236
2	1.767	6.310	43.546	1.767	6.310	43.546
3	1.564	5.586	49.132	1.564	5.586	49.132
4	1.466	5.237	54.369	1.466	5.237	54.369
5	1.317	4.702	59.071	1.317	4.702	59.071
6	1.246	4.450	63.521	1.246	4.450	63.521
7	1.204	4.300	67.821	1.204	4.300	67.821
8	1.118	3.992	71.813	1.118	3.992	71.813
9	1.049	3.745	75.558	1.049	3.745	75.558
...	...	...	...			
...	...	...	...			
28	0.216	0.770	100			

### Reliability and validity testing

The questionnaire measured 33 items and was divided into 9 variables. The Cronbach’s alpha was 0.937, and the Cronbach’s alpha coefficients of each latent variable reached the basic criterion of higher than 0.7, showing that the questionnaire had good reliability.

We performed confirmatory factor analysis (CFA) in Amos. In Table 3, the standardized factor loading of each item all higher than 0.5, and the standard error values (S.E.) were all less than 0.5, showing that every item could organize its dimension, which is consistent with the results of exploratory factor analysis, indicating good validity of the variables in the questionnaire.

**Table 3:** Reliability and validity measurement scales.

Variable	Item	Standardized factor loading	S.E.	*t*-value	CR	AVE
User involvement	UI3	0.764	0.067	11.403	0.844	0.644
	UI2	0.834	0.067	12.448		
	UI1	0.825	0.069	11.957		
Information quality	IQ3	0.752	0.072	10.444	0.826	0.614
	IQ2	0.799	0.072	11.097		
	IQ1	0.798	0.072	11.083		
Service quality	SQ3	0.783	0.070	11.186	0.822	0.605
	SQ2	0.804	0.068	11.824		
	SQ1	0.745	0.071	10.493		
Platform reputation	PA3	0.786	0.064	12.281	0.834	0.627
	PA2	0.84	0.064	13.125		
	PA1	0.745	0.065	11.462		
Emotional support	ES3	0.816	0.061	13.377	0.839	0.635
	ES2	0.77	0.063	12.222		
	ES1	0.805	0.062	12.984		
Perceived usefulness	UPU1	0.778	0.061	12.754	0.853	0.659
	UPU2	0.779	0.062	12.565		
	UPU3	0.796	0.061	13.049		
User trust	UT1	0.773	0.063	12.270	0.839	0.635
	UT2	0.771	0.062	12.435		
	UT3	0.768	0.065	11.815		
Value co-creation behavior	UVCB4	0.74	0.061	12.131	0.867	0.621
	UVCB3	0.716	0.061	11.738		
	UVCB2	0.715	0.061	11.721		
	UVCB1	0.755	0.063	11.984		
Continuance intention	CUW3	0.652	0.093	7.011	0.802	0.575
	CUW2	0.767	0.094	8.160		
	CUW1	0.784	0.092	8.522		

Combination reliability (CR) is a criterion for determining the basic reliablity of a model, indicating if all measurements in every latent variable consistently explain it. The combination reliability of each variable in Table 3 was higher than 0.7, proving that all measurements in every latent variable could consistently explain it.

The average variance extracted (AVE) value is commonly used to show the convergent validity of a scale and indicates how much of the variance explained by the latent variable comes from measures error. The larger the AVE value, the smaller the relative measurement error and the higher the percentage of variance influenced by the latent variable. Table 3 shows AVE in our questionnaire variables were all above 0.5, which is standard minimum value to indicate good convergent validity.

### Model adaptation indicators

**Figure 2:**
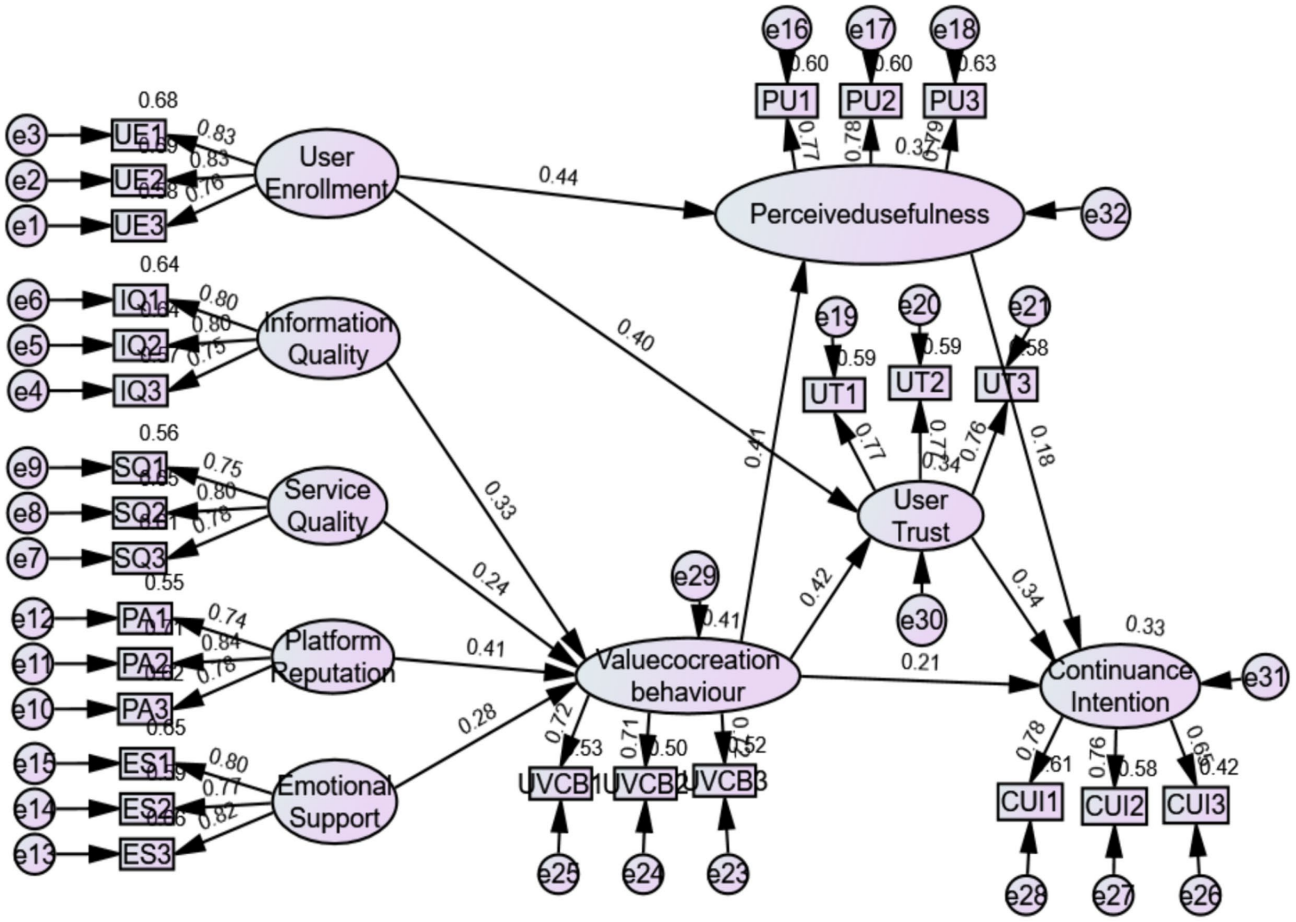
Structural equation model path analysis diagram.

According to the Table 4 below, all indicators met the universal standard, indicating that the CFA model built in this article was effective and well-matched with the recovered data. Figure 2 shows the results of a path analysis to determine the associations between variables in the model. Information quality (*β* = 0.33, *p* < 0.05) and service quality (*β* = 0.24, *p* < 0.05) had an obvious impact on UCVB, thus H1 and H2 are demonstrated. Additionally, platform reputation (*β* = 0.41, *p* < 0.05) and emotional support (*β* = 0.28, *p* < 0.05) had a significant positive impact on continuance intention, demonstrating hypotheses H3 and H4.

**Table 4:** Fitting degree of index model.

Index	*χ*^2^/df	RMSEA	GFI	AGFI	IFI	TLI	CFI	NFI
Reference value	<3	<0.08	>0.9	>0.9	>0.9	>0.9	>0.9	>0.9
Observations	2.774	0.035	0.917	0.897	0.973	0.9968	0.973	0.973

### Test of mediation effect

We adopted a bias-corrected nonparametric percentile bootstrap method based on the Hayes process macro. We used Model 4 in the SPSS plugin, with results shown in Table 5. The total effect value of user value co creation behavior on the willingness to continue using is 0.387, with a 95% confidence interval of [0.301–0.473], which does not pass through 0, indicating that the total effect is valid. The direct effect value is 0.244, with a 95% confidence interval of [0.153–0.334], which does not pass through 0, indicating that the direct effect is valid. The effect value of the indirect path mediated by trust is 0.143, with a 95% confidence interval of [0.095–0.196], which does not pass through 0, indicating that the mediating effect is valid. Trust plays a significant mediating role between value co creation behavior and willingness to use; thus, hypothesis H5 is demonstrated. The effect of the indirect path mediated by user perceived usefulness is 0.125, with a 95% confidence interval of [0.077, 0.178]. User perceived usefulness takes a significant mediating part between value co-creation behavior and sustained use intention, so hypothesis H6 is confirmed (Tables 4, 5).

**Table 5:** Bootstrap mediation effect test results.

Effect Relationship	Route	Value	se	t	p	95%CI	
						LLCI	ULCI
Total effect	Value co-creation behavior–continuance intention	0.512	0.044	11.636	0.001	0.301	0.473
Direct effect	Value co-creation behavior–continuance intention	0.244	0.046	5.304	0.001	0.153	0.334
Indirect effect	Value co-creation behavior–user trust–continuance intention	0.143	0.025	–	–	0.095	0.196
	Value co-creation behavior–perceived usefulness–continuance intention	0.125	0.026			0.077	0.178

### Tests of moderating effects

**Figure 3:**
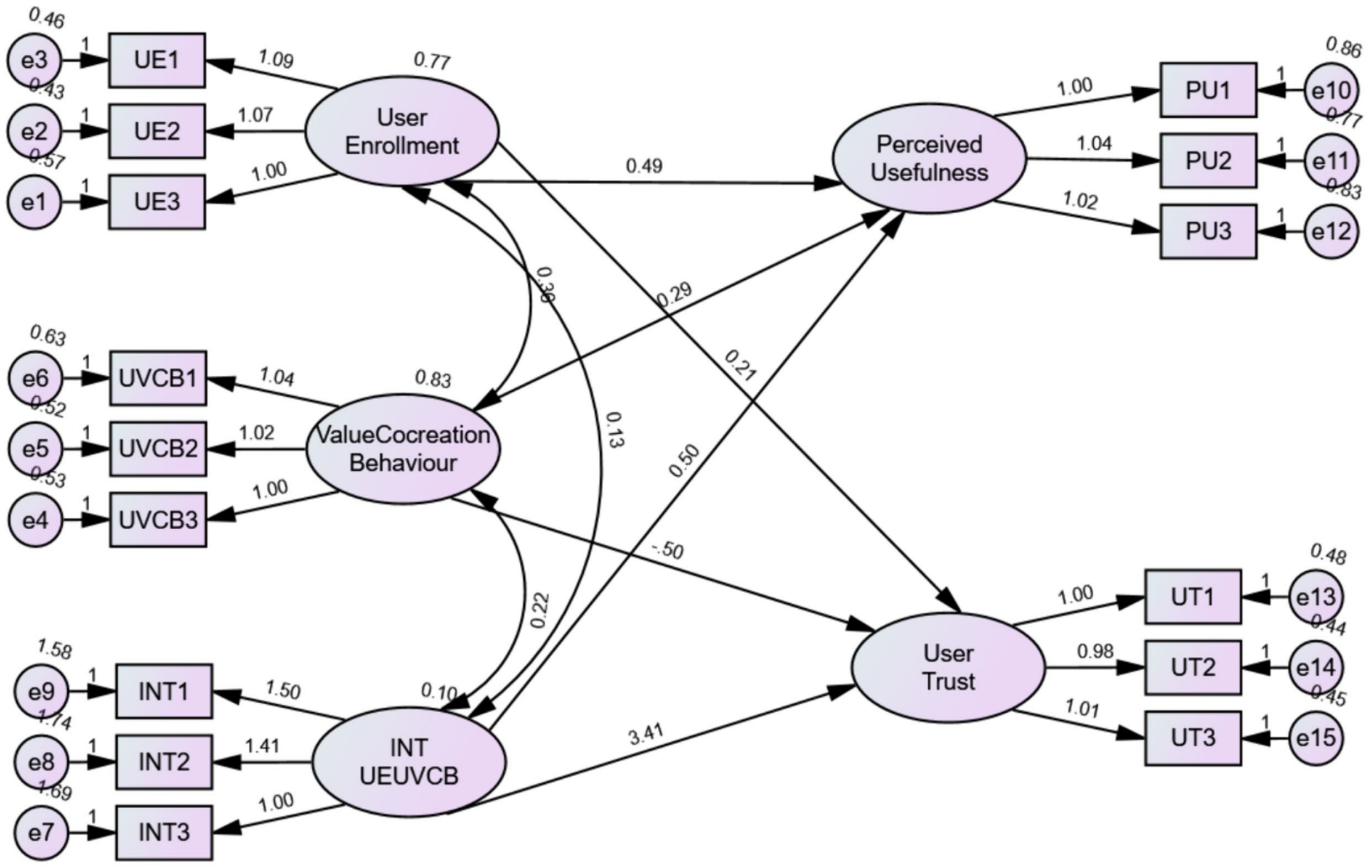
Moderation effect path analysis.

In order to decrease the measurement errors, we add the interaction item of User enrollment and UVCB into the SEM model and estimated it (Figure 3). The data were showed in Table 6. The interaction item of User enrollment and UVCB has significant impact on user trust (*t* = 3.27, *p* < 0.05) and perceived usefulness (*t* = 1.695, *p* < 0.05). Therefore, user enrollment positively moderating the association between the independent variable and the dependent variable, and H5, H6 is proved.

**Table 6:** Moderation effect test.

		Estimate		S.E.	C.R.	*p*
Perceived_Usefulness	<---	User_Enrollment	0.489	0.061	8.063	***
Perceived_Usefulness	<---	Value Co-creation_Behavior	0.286	0.071	4.048	***
Perceived_Usefulness	<---	INT_UEUVCB	0.495	0.223	2.226	**
User_Trust	<---	User_Enrollment	0.213	0.154	1.387	0.166
User_Trust	<---	Value Co-creation_Behaviour	−0.503	0.215	−2.337	**
User_Trust	<---	INT_UEUVCB	3.408	0.943	3.613	***
UE3	<---	User_Enrollment	1			
UE2	<---	User_Enrollment	1.071	0.071	15.12	***
UE1	<---	User_Enrollment	1.093	0.073	15.062	***
UVCB3	<---	Value Co-creation_Behaviour	1			
UVCB2	<---	Value Co-creation_Behaviour	1.02	0.071	14.43	***
UVCB1	<---	Value Co-creation_Behaviour	1.038	0.074	14.063	***
INT3	<---	INT_UEUVCB	1			
INT2	<---	INT_UEUVCB	1.411	0.395	3.569	***
INT1	<---	INT_UEUVCB	1.504	0.407	3.699	***
UT1	<---	User_Trust	1			
UT2	<---	User_Trust	0.984	0.066	14.971	***
UT3	<---	User_Trust	1.007	0.067	15.028	***

**p<0.01; ***p<0.001.

## Conclusion

In this paper, our analysis confirms the validity of the eight hypotheses. Information quality, service quality, corporate reputation, emotional support can positively influences UVCB. That is to say, users are more willing to interact and co-create value with enterprises if the information and services are of high quality, which is consistent with previous research findings (27, 28). Corporate reputation and emotional support also significantly affect UVCB; further, users provide feedback to the platform through dialog and negotiation when they believe that the digital health resources are reliable and the enterprise can provide them with a sense of belonging and satisfaction (19). Our analysis also demonstrates that user trust and perceived usefulness mediate the association between UVCB and their continuance intention. User behaviors that enhance their trust and perceived usefulness of digital health resources promote their long-term and continued use of those resources (31). Moreover, our study confirmed that user enrollment can positively moderate the association between UVCB and both user trust and perceived usefulness. As such, greater user involvement results in stronger trust in and greater perceived usefulness of digital health resources. Also, digital health resources improved efficient interaction and communication between doctors and patients; effectively improve users’ acceptance of treatment plans; and reduce doctor–patient conflicts.

## Discussion

Based on the conclusion, we proposed some strategies to enhance users’ willingness to sustain the use of digital health resources. Referencing the central path of the ELM, the companies should improve the quality of services and information to promote the value co-creation behaviors of users. Internet hospitals should provide users with sufficient information about doctors and hospitals before consultation to reduce medical disputes caused by information asymmetry (52). Auditing of information should increase to avoid the emergence and spread of false and inaccurate health information (53).

Regarding service quality, the online consultation mode is prone to misdiagnosis and omission due to unreliability of the equipment network and the complexity of patient conditions. Internet hospitals should implement the first consultation offline, and use the online interface only for examination reports and follow-up. More excellent doctors should be recruited to improve the professional level of online consulting services, and supervision should be strengthened (2). Enterprises can also promote health knowledge and increase the popularity of their platforms through short health videos, audio, graphics, and other forms of digital information (54).

For users who process information by the peripheral path, we found that both good platform reputation and emotional support facilitate users’ value co-creation behavior. Enterprises could improve their reputation using blockchain technology to strengthen data security so that users do not have to worry about privacy leakage. Enterprises should also optimize the interface design so that each functional partition is simple and easy for users to operate. The establishment of a community where users can help with each other online brings them emotional support and promotes users’ continuance intention. The warmth of an online community can strengthen users’ awareness of the provider and improve its reputation. The government should provide appropriate policy support to reduce the tax pressure on digital health service providers. At the same time, the government can promote users’ continuance intention by formulating eHealth education programs and organizing health-themed activities that enhance users’ online literacy and improve their ability to make health-related decisions.

The most important implication and contribution of this study is that we have investigated the current status and problems of the digital health resources and we proposed the suggestions to the companies and government. Also, we established a comprehensive structural equation model, which can provide the guidance and experiences for the future research. Our proposals can be used to promote the quality of the health digital resources and boost health industry. In this way, the residents’ health status and the health outcome will be improved.

The study has some shortages that could be expanded and improved in future. Firstly, the sampling data came from the region of the Yangtze River Delta, where economic conditions are good and users have relatively higher health literacy and higher acceptance of digital health resources. The geographic scope of the sample collection should be expanded in future studies to make the sample richer and more diverse. Second, this study collected cross-sectional sample data from only one period of time. In the future, we should consider collecting data at different periods, and conduct a dynamic qualitative comparative analysis to present the research results more completely and macroscopically.

## Data availability statement

The original contributions presented in the study are included in the article/supplementary material, further inquiries can be directed to the corresponding author.

## Ethics statement

The studies involving humans were approved by Ethics Committee of Wenzhou Medical University. The studies were conducted in accordance with the local legislation and institutional requirements. The participants provided their written informed consent to participate in this study.

## Author contributions

CF: Software, Resources, Project administration, Methodology, Investigation, Formal analysis, Data curation, Conceptualization, Writing – review & editing, Writing – original draft. HZ: Writing – review & editing, Writing – original draft, Investigation, Formal analysis, Data curation, Resources. WW: Writing – review & editing, Visualization, Supervision, Software, Investigation, Conceptualization. LJ: Writing – review & editing, Validation, Supervision, Investigation, Formal analysis, Conceptualization. YX: Writing – review & editing, Investigation, Data curation, Conceptualization. HY: Writing – review & editing, Validation, Supervision, Resources, Project administration, Methodology, Funding acquisition.
